# Selections and Abstracts

**Published:** 1898-03

**Authors:** 


### *SELECTIONS AND ABSTRACTS.*

### Progress of Serum-Therapy.

A committee appointed last April by the Richmond (Virginia) Academy of Medicine to investigate and report upon the progress of serum-therapy have just submitted their report, of which the following is part:

Diphtheria.—The history of the development of the antitoxin treatment of diphtheria is so well known, and its acceptance now so universal, that it would be useless, even if the limits of this report rendered it possible, to go fully into details on these points. Even at the time that your committee was appointed it could be said that a sufficiently extensive trial of the antitoxin treatment of diphtheria had been made to remove the fears which many had entertained as to its action, and to place the remedy in an assured position as one of inestimable worth.

During the past year statistics from many and varied sources have appeared, all tending still further to increase the confidence of the profession in the remedy, and to demonstrate the saving of life which it has accomplished. Many who were sceptical have been convinced by the unanswerable argument of statistics whose authenticity could not be questioned, until to-day it may be said that but few physicians, indeed, who, having given the subject careful consideration, do not accord to the remedy even more value than was at first claimed for it by its staunchest advocates.

Only two sets of these statistics will be referred to, not because they are more favorable than others, but on account of the large number of cases recorded in them, and because they represent such diversity in the class of cases treated.

The report of the committee of the American Pediatric Society, presented at its last meeting, includes 1,704 cases of laryngeal diphtheria which occurred in the practice of 422 physicians in the United States and Canada. In this report the following points are among the most prominent: Before the use of antitoxin it was estimated that 90 per cent. of laryngeal diphtheria cases required operation, whereas now, with the use of antitoxin, only 39.21 per cent. require it. The mortality in the whole series of 1,704 cases was 21.12 per cent. (360 deaths). In the non-operated cases the mortality was 17.18 per cent. (178 deaths). The mortality in the operated cases (27.24 per cent.—182 deaths) shows even more remarkable results. Before the use of antitoxin only 27 per cent. *recovered;* now only 27.24 per cent. *die.*

The other report to which reference will be made is the "Second Report of Medical Superintendents upon the Use of Antitoxic Serum in the Treatment of Diphtheria in the Hospitals of the Metropolitan Asylum's Board during the year 1896." (London.) In these hospitals during 1896 antitoxin was used in 71.3 per cent. of all cases of diphtheria, the remedy not being employed in moribund, mild, or doubtful cases. The total death-rate during 1896 (under antitoxin) was 20.8 per cent.; that of 1894 (without antitoxin), although then considered remarkably low, was 29.6 per cent. This represented a saving of 365 lives. It is well known, however, that only during the early days of the disease does antitoxin exert its full beneficial effect; and hence, while the above figures show a difference in the total death-rate of only 8.8 per cent., the difference in cases treated on the first day was 17.8 per cent.; on the second day, 14.2 per cent.; on the third day, 11.7 per cent.; on the fourth day, 9.1 per cent., and on the fifth day or later, 6.2 per cent. Laryngeal cases were attended with a mortality of 62 per cent. in 1894; 29.6 per cent. in 1896. Operated laryngeal cases had a death-rate of 70.4 per cent. in 1864; 41 per cent. in 1896. It is the opinion of the superintendents that there has been no reduction in the frequency of complications of the disease as a result of antitoxin treatment, except in the case of nephritis, which occurs less often. In fact, it would seem as if the other complications occur even more frequently than formerly. This, however, is only apparent, and is due to the closer observations which are now made, and, even more, to the fact that many severe cases, which would have died under other treatment, now recover, and these are naturally more prone to develop complications.

In this report the general results of antitoxin treatment are summed up as follows: 1. Diminution of faucial swelling; 2. Lessening of irritating and offensive discharge from the nose; 3. Limitation of extension of membrane; 4. Earlier separation of exudate; 5. Limitation and earlier separation of membrane in laryngeal cases; 6. Improvement in general condition and aspect of patients; 7. Prolongation of life, in fatal cases, to an extent not obtained with former methods of treatment.

It has been claimed by those who refuse to recognize the value of diphtheria antitoxin, that the favorable results shown in the statistics of the past few years are due to other factors than the employment of antitoxin. By some it is held that the type of the disease has become milder; others that since the wide-spread application of bacteriologic diagnosis, cases are now called diphtheria which were formerly not so classified; or, again, that moribund cases and cases treated after the fifth day of the disease are excluded in many of the statistics. The first report to which reference has been made above answers fully the first objection, since it deals with only laryngeal cases, and laryngeal diphtheria can never be considered mild. In the report of the Metropolitan Asylum's Board Hospitals mild as well as moribund cases were not injected. As regards the influence of bacteriologic diagnosis, it is a fact which no one acquainted with the subject will dispute, that the number of cases which would formerly have been considered diphtheria, but which are now excluded from the statistics by bacteriologic investigations, far exceed those in which the reverse is true.

While the above are strong replies to the criticisms of the opponents of antitoxin, we are indebted to Park* for a table of statistics against which none of the usual objections can be urged. This table, compiled from the official records of Berlin, Paris and New York, shows the absolute death-rate per 100,000 inhabitants in these cities from diphtheria and croup from 1886 to 1897 inclusive. There is here no room for asserting that the statistics have been twisted to favor any plan of treatment, that any special class of cases has been excluded or included, and yet the reduction in mor-

* Park, Wm. H.: Contributions of Bacteriology to Therapeutics. Wesley M. Carpenter, Lecture for 1897.

tality in all three of these cities since the introduction of antitoxin is remarkable and too uniform to be the result of mere coincidence. This table is of such interest that it is here appended :

ABSOLUTE DEATH-RATE FROM DIPHTHERIA AND CROUP PER 100,000 POPULATION.

| YEAR. | BERLIN. | PARIS. | NEW YORK. |
|---|---|---|---|
| 1886 | 125.7 | 73.2 | 187.5 |
| 1887 | 100.7 | 76.9 | 206.6 |
| 1888 | 76.1 | 83.7 | 167.7 |
| 1889 | 85.6 | 79.9 | 146.2 |
| 1890 | 102.0 | 77.5 | 110.6 |
| 1891 | 67.5 | 63.0 | 118.7 |
| 1892 | 92.9 | 63.6 | 123.3 |
| 1893 | 100.8 | 51.4 | 145.5 |
| 1894 | 86.7 | 40.7 | 158.5 |
| 1895 | †59.7 | 17.7 | 105.2 |
| 1896 | 30.9 | 17.6 | 91.3 |
| *1897 | 26.4 | 17.2 | 86.4 |

As regards actual advances in the antitoxin treatment of diphtheria, the chief of these seems to be the production of more potent and trustworthy serums and the attention to details in its manufacture, whereby its efficacy has been increased and many of its objectionable features diminished. Rashes and joint symptoms following its use are now somewhat less frequent than formerly.

The preparation of dried serum has not yet been brought to a sufficient degree of perfection to supplant the ordinary product, while "we have no more hope than we had five years ago of separating antitoxin completely from the horse serum." (Park.)

During the past few months the Health Department of New York city has been testing the comparative frequency of rashes after the use of filtered and unfiltered serums. Your committee is indebted to Dr. Wm. L. Sommerset, Resident Physician Willard Parker Hospital, for the following statement, which is an approximate one, of the results obtained : Previous to the employment of filtered serum, rashes occurred at the Willard Parker Hospital in from 25 to 30 per cent. of all cases; since the use of filtered and unfiltered serum in parallel cases, the percentage of rashes where

* Last quarter of year estimated.

† General use of antitoxin commenced.

filtered serum was injected has been reduced to about 15 per cent., while it has risen to about 40 per cent. where the unfiltered product was employed. The higher percentage of rashes in the latter class of cases than formerly is due to the fact that the unfiltered serum used in these cases contained the residue from the filtered portion. It would thus appear that the production of rashes is caused largely by some constituent of the serum which is incapable of passing, or passes only in small amounts, through unglazed porcelain.

The use of antitoxin as a prophylactic measure has been steadily gaining ground, and with the production of a serum from which all objectionable features have been eliminated, its use in this direction will doubtless become even more popular; though, from the temporary nature of the immunity affected, it must remain a measure to be adopted only in the presence of epidemics or in cases where exposure has undoubtedly occurred.

YELLOW FEVER.—In 1854 and 1855, inoculation, as a preventive measure, was made use of in Havana during an epidemic of yellow fever. In 1887, this idea was again introduced and followed up very thoroughly in Brazil. But in neither instance were the results satisfactory. In 1892, Domingo Freire introduced a diluted virus derived from the micrococcus xanthogenicus, which he held to be the etiological factor in the development of yellow fever. This diluted virus was advocated as a preventive inoculation by Dr. Belinger, of San Francisco, Dr. J. McFadden Gaston, of Atlanta, and others during 1893 and 1894; but the results have been disappointing. About the same time, 1893, Dr. A. S. Ashmead recommended "Murray's immunizing method" as follows: Inoculate with the blood serum of a partially immune subject (negro), and inoculate a second time with perfectly immune blood serum of a white subject who has had yellow fever. Before inoculation, however, as frost always modifies the virus, let the infected serum be first exposed to frost. Follow at once with a second inoculation of immune blood serum." Disappointments likewise followed the use of this method.

In July, 1897, Prof. G. Sanarelli, of the University of Montevideo, isolated and cultivated a bacillus which he considers to be the specific organism of yellow fever. Probably it is the same bacillus as that formerly described by Surgeon-General Sternberg. Possibly both may ultimately be proven to be secondary invaders. However, Sanarelli has been occupying himself since last summer in the securing of a protective or curative serum, about which most encouraging reports have been already made. And yet scarcely is the hope born that at length we have a protective or curative agent with which to meet yellow fever before adverse reports are coming in to indicate that we must wait and see.

TUBERCULOSIS.—The medical world was startled in 1890 by the announcement that Koch had discovered a remedial agent for phthisis. This announcement was hailed with joy, and it was immediately put to the clinical test, but its virtues, so ably set forth by its discoverer, soon began to minimize, and finally it fell into disrepute as a curative remedy. Since the introduction of tuberculin, several serums have been brought forward, and have been tried with varying success by many physicians.

Among the most popular of these preparations is the serum of Prof. E. Maragliano of Genoa, obtained from the dog, the ass, and the serum. When treatment was begun as late as the formation of cavities in the lungs, he claims a *cure* of 7.76 per cent. In non-febrile tuberculosis, his successes have amounted to nearly 100 per cent. of recoveries. He recommends that 1 c. m. of the serum should be the dose injected subcutaneously every second day. In febrile forms the dose may be increased for several days—5 to 8 days—to 5 and even 10 c. m. Such are the contradictory reports as regards successful use of Maragliano's serum by different doctors that it is difficult to come to a fixed opinion on the subject.

What has been said in general of Maragliano serum applies in the main to the published results of the use of the antitubercle serum introduced in 1895 by Dr. Paul Paquin of St. Louis. That it is useful when administered with other remedies is more than probable.

Early in 1896, Koch introduced what he called T. R. Tuberculin, and this was followed by encouraging reports of its use. But it was not long before the process of its manufacture was found to be faulty in that, notwithstanding the centrifugation, it was discovered that in a large number of the preparations on the market tubercle bacilli remained in the fluid. This being recognized as an error of manufacture, it has been withdrawn from market—certainly until the fault of its manufacture can be remedied.

Antiphthisin is a sozalbumin, introduced some years ago by Klebs, which he regards as the germicidal part of tuberculin. Von Ruck "attests its absolute safety, and considers that it has curative properties."

But the early disappointments in practice of the serum treatment of tuberculosis have made the profession skeptical as to the remunerative value of any and all such methods of treatment; and yet it is evident to the non-skeptical, who reviews the experience of unbiased practitioners, that it is probable that whatever may be found curative of tuberculosis, one of the measures to be used will be perfection of some of the antitoxins so called. It is the opinion of many that the scientific worker is getting in the neighborhood of the real remedy, and is probably knocking at a door of the house in which the truth is to be found.

TYPHOID FEVER.—Chautemesse and Widal found, in 1888, that susceptible animals could be rendered immune by inoculating them with a culture of the typhoid organisms which had been rendered sterile by heat.

Following this line of investigation, serum-therapy has been employed in typhoid fever. However, with the most persistent use of this substance, the results in this disease have been unsatisfactory.

SCARLET FEVER.—An antistreptococcic serum has been employed in this affection, and while it does not seem to have any efficacy in the scarlet fever itself, yet, from a certain number of observations made by Roux, it appears to have a favorable influence on the complications due to the streptococcus, and which are so common in this malady.

TYPHUS FEVER.—In a prison at Bougie, from November 25th to December 12th, forty cases occurred, with two deaths. Serum-therapy was commenced December 12th, the serum being obtained from patients who had recovered. From 2 to 6 c.c. were injected into each of 39 c., and only one died. Legrain noted fall of temperature, improvement in pulse, disappearance of fever, and that severe cases soon became mild in type.

Although this disease is rare, owing to improved hygienic measures, yet, from the favorable report of serum in its treatment, it is to be hoped that the rate of mortality will be kept lowered in those instances where disease gains a foothold.

RABIES.—Tizzoni and Cattani reported that serum of animals rendered immune to hydrophobia has the power of conferring immunity and arresting the disease when already developed. So far no trial has been made in man. If successful here, its value will surpass the treatment of Pasteur, in that the latter may produce immunity, but cannot cure the disease if once developed.

LEPROSY.—Carrasquilla treated a number of cases of leprosy with serum, and in fifteen cases of the anesthetic type secured a remarkable degree of success. Medina, commenting on this treatment, sums up as follows :

"The sensation is restored more or less rapidly according to the extent and gravity of the lesions of the peripheral nervous system. Distinct patches lose their abdominal color without disappearing entirely. Edema subsides rapidly in some cases, slowly in others. The skin shrinks and resumes its physiological condition after the absorption of the edema. The tubercular variety shrinks, breaks down, and disappears by absorption, desquamation, or suppuration, leaving traces of their existence at the affected spots. Ulcers, after having presented copious purulent secretions, rapidly clear off and are covered with healthy skin ; scars from former suppurative lepromata fade and tend to assume the same level as the surrounding skin. Ulcerated mucous membranes fade like the skin and recover sensibility, while the tubercules break down. The face loses its leonine appearance. Lastly, the patient recovers his appetite, the ability to sleep, and the mental state improves."

SARCOMA AND CARCINOMA.—Varying success has attended the use of bacterial products in inoperable malignant disease. Coley, who has done a great deal of work in this class of cases, secured greater success with the treatment in sarcoma than in carcinoma. The conclusions of the New York Surgical Society, which may be considered as a conservative survey of the efficacy of this treatment in malignant disease, are as follows (*Annals of Surgery*, July, 1896):

"(1) The danger to the patient from the treatment is great.

"(2) The alleged successes are so few and doubtful, the most that can be said for the treatment is that it may offer a slight chance of improvement.

"(3) Valuable time has been lost in operable cases by giving the treatment a trial before operating.

"(4) The method should be absolutely confined to inoperable cases."

SYPHILIS.—Richet and Haricourt were the first to try injections of serum in the treatment of syphilis. They report two cases, one resulting in a cure; the other showed marked signs of improvement—the ulcerated surface decreasing four-fifths in size. Pellizzari and Neuman have employed blood in its treatment, but no satisfactory results were obtained.

STREPTOCOCCIC INFECTIONS.—In *erysipelas* Marmoreck used the antistreptococcic serum in 413 cases in which the mortality was 3.87 per cent. against 5 per cent. prior to the use of serum. In 165 cases of erysipelas alone the mortality was 1.2 per cent. As a result of the use of the serum he noticed an improvement in the general condition, fall of temperature, and local improvement.

*Puerperal fever* has been treated by this plan with a certain degree of success. It has not proven as useful in this disease as there was reason to expect at the first mentioning of the subject.

The serum has also been used with success in acute hemorrhagic septicemia, erysipelas neonatorum, acute genital septic peritonitis, purulent cellulitis of the face, cerebro-spinal meningitis.

PNEUMONIA.—Klemperer, in 1892 and 1893, published his researches as to the treatment of serum-therapy in six cases with good results. Mosny, Bonone, Emmerich, and Pansini, following up this idea, obtained practically the same results as Klemperer. On the other hand, Hughes and Carter, in 1894, reported the injection of the serum of patients just recovered from pneumonia in ten cases of this disease, and they had no reason to consider the treatment efficacious.

CHOLERA.—Many experiments have been made in cholera, chiefly as inoculations, and these mostly in animals until recently. During an epidemic of this dread malady in Spain, 50 per cent. of those attacked died. After inoculation was used, the rate of mortality was reduced to 25 per cent.

INFLUENZA.—The bacteria of influenza was discovered in 1892. Rabbits were inoculated with cultures, and after they had been proven immune, the blood serum was used on men suffering from influenza with varying success.

The serum treatment has been extended considerably in its scope, so that at the present time there are but few of the more infectious diseases, to which mankind is liable, that have not been considered by the experimental bacteriologist. In addition to the diseases above referred to may be mentioned the *bubonic plague,* anthrax, alcoholism, acute delirious mania, glanders, pleuro-pneumonia, foot and mouth disease, chicken cholera, hog cholera and bites of reptiles, as other diseases in which serum-therapy has been employed.

TETANUS.—Behring and Kitasato were the first to produce in the blood of animals a distinctly potent and valuable antitoxic substance. In 1892 Dr. Rudolph Schwarz reported the first success with tetanus antitoxin in man. Cases of cure have been reported from time to time which gradually increase the per cent. of recoveries. It may be very properly asked why the success with serum in this disease is not so favorable as that in diphtheria—the two antitoxins being so nearly identical in their efficacy as agents for immunization? The answer may be found "in the manner" in which "the two infections begin and progress." * The tetanus affection advances for from five to ten days undetected in the wound, the toxins develop unnoticed, cause their deleterious effect, and only after general poisoning has occurred is the disease detected.

The diphtheria infection starts on the surface, and before its toxins are fully developed and absorbed, it is diagnosticated by the appearance of its exudate, the husky voice, or the nasal obstruction. At this time we could also arrest the tetanus infection. If we allow this time to pass by, then, as in tetanus, after the constitutional poisoning has taken place, the antitoxin is usually of no avail.

* The Wesley M. Carpenter Lecture for 1897. By Wm. H. Park, M.D.

An improvement in the form of tetanus antitoxin is the solid variety. It is claimed that this preparation can be kept indefinitely, and is ready for use at any time by simply dissolving it in water.—DRS. ARTHUR JORDAN, LANDON EDWARDS AND E. C. LEVY, *Committee.*

---

### THE TREATMENT OF PULMONARY TUBERCULOSIS BY THE HYPODERMIC USE OF A COMPOUND SOLUTION OF IODINE.

Dr. Chas. W. Ingraham, of Binghamton, New York, believes (*N. Y. Medical Journal,* October 23, 1897) that "in iodine we have a powerful antitubercular remedy, though, unfortunately, on account of its tendency to irritate the mucous membranes of the stomach, its continued use cannot be tolerated by the average consumptive invalid. It is customary to find the stomach of the pulmonary invalid already weakened by the effect of the tuberculous disease, and when we attempt to administer a remedy which tends to increase the existing irritation, disastrous results must ensue. Hence, though the effect of iodine might be strongly indicated, the administration of the drug by the stomach is out of the question in nine cases out of ten.

"The formula of my compound solution of iodine was perfected in the latter part of 1892, and now, after five years' use in the treatment of all forms of phthisis, it has proved so thoroughly satisfactory and generally applicable that I have made no alterations or additions to the original formula. I should state that the compound is prepared for hypodermic use only, sterilized oil being the solvent used. Each drachm of the compound represents approximately the following:

| | |
|---|---|
| Iodine (chemically pure) | gr. $\frac{1}{2}$. |
| Bromide (chemically pure) | gr. $\frac{1}{14}$. |
| Phosphorus (chemically pure) | gr. $\frac{1}{100}$. |
| Thymol (chemically pure) | gr. $\frac{2}{3}$. |
| Menthol (chemically pure) | gr. $\frac{2}{3}$. |

"It must be borne in mind that the compound is not a simple mixture of the ingredients herein mentioned, but that the properly prepared product is the result of a series of definite chemical reactions. The characteristics of the compound are as follows: It is of a bright cherry color, distinctly transparent, and of a slight aromatic odor. It will keep indefinitely without undergoing any change if kept protected from light and heat.

"My report in the *Bulletin* two years and a half ago shows nearly ninety per cent. of cures of all cases of pulmonary tuberculosis treated in the first stages of the disease, while the percentage of recoveries in the second stage of the disease was nearly fifty. These percentages were based upon twenty-one cases treated in the first stage and fourteen cases treated in the second stage—a total of thirty-five."

---

### SOME "DON'TS" ABOUT HEART DISEASE.

*Don't* feel called upon to give digitalis as soon as you hear a murmur over the heart. Study and treat the patient not the murmur.

*Don't* conclude that every murmur indicates disease of the heart.

*Don't* forget that the pulse and general appearance of the patient often tell more than auscultation.

*Don't* neglect to note the character of the pulse when you feel it. Possibly you may look at the tongue to satisfy the patient; feel the pulse to instruct yourself.

*Don't* think every systolic murmur at the apex indicates mitral regurgitation; every systolic murmur at the aortic interspace, aortic stenosis. The former may be trivial; the latter may be due to atheroma of the arch of the aorta.

*Don't* say every sudden death is due to heart disease.

*Don't* forget that the most serious diseases of the heart may occasion no murmur. A bad muscle is worse than a leaky valve.

*Don't* examine the heart through heavy clothing.

*Don't* give positive opinions after one examination.—*Philadelphia Medical Journal.*

---

According to the *Baltimore Sun,* smallpox this winter appears to have been most prevalent in the vicinity of Birmingham, Ala., where many thousand negro coal-miners are employed, and from thence disseminated elsewhere. The great difficulty in the way of preventing the spread of the disease was the refusal of the negroes to be vaccinated. Finally, the local authorities appealed to the supervising Surgeon-General of the Marine Hospital Service. Dr. Wyman immediately dispatched Assistant-Surgeon Magruder, with thirty-odd assistants, to the infected territory near Birmingham. At the suggestion of the United States officials, the mine-owners and superintendents joined in a notice to the miners that employment would no longer be given unless they consented to be vaccinated. The Surgeon-General has just received a report from Dr. Magruder that in a little over two weeks more than 25,000 had been vaccinated by his assistants, over 14,000 houses inspected, and the disease is being rapidly stamped out. When Alabama is through with Dr. Magruder and his corps, by request of the State Board of Health he will proceed to Tennessee and make a wholesale vaccination in all infected places in that State.—*Phila. Med. Journal.*

---

### GRAY'S GLYCERIN TONIC.

℞ Fluid extract of cinchona............................ʒ 3.

Diluted phosphoric acid............................ʒ 10.

Glycerin............................................℥ 4.

Sherry wine.........................................℥ 10½.

The above was the formula of the "glycerin tonic" used so much by the late Dr. John P. Gray, of the New York State Lunatic Asylum.—*Druggists' Circular.*

---

### A PILL FOR MIGRAINE.

The *Journal des practiciens* for January 29th gives the following formula:

℞ Quinine valerianate..............................1½ gr.

Caffeine citrate..................................¾ gr.

Extract of Indian hemp..........................$\frac{15}{100}$ gr.

M. Two or three such pills to be taken daily.

—*N. Y. Med. Journal.*

